# Toll-Like Receptors Gene Polymorphisms in Autoimmune Disease

**DOI:** 10.3389/fimmu.2021.672346

**Published:** 2021-04-26

**Authors:** Yingchi Zhang, Jia Liu, Changlun Wang, Junxian Liu, Wei Lu

**Affiliations:** Department of Neurology, The Second Xiangya Hospital, Central South University, Changsha, China

**Keywords:** gene polymorphisms, autoimmune disease, toll-like receptors, pathogenesis, treatment

## Abstract

Toll-like receptors (TLRs) are important initiators of the immune response, both innate and acquired. Evidence suggests that gene polymorphisms within TLRs cause malfunctions of certain key TLR-related signaling pathways, which subsequently increases the risk of autoimmune diseases. We illustrate and discuss the current findings on the role of Toll-like receptor gene polymorphisms in numerous autoimmune diseases in this review, such as type 1 diabetes mellitus, Graves’ disease, rheumatoid arthritis, systemic lupus erythematosus and multiple sclerosis. The study of genetic variation in TLRs in different populations has shown a complex interaction between immunity and environmental factors. This interaction suggests that TLR polymorphisms affect the susceptibility to autoimmune diseases differently in various populations. The identification of Toll-like receptor gene polymorphisms can expand our understanding of the pathogenesis of autoimmune diseases, which will subsequently guide effective medical management and provide insight into prognosis and advanced treatments.

## Introduction

Autoimmune diseases develop when the immune response is misdirected to the host (1). These diseases range from organ-specific diseases (in specific tissues, antibodies and T cells react to self-antigens) to organ-nonspecific or systemic diseases (characterized by reactions to antigens distributed in multiple tissues) (2). Regarding the mechanism of derailed immune responses in the pathogenesis of autoimmune diseases, abnormal processes associated with the immune system are involved in the initiation and perpetuation of disease (3). Recent epidemiological studies have shown a high prevalence of autoimmune diseases affecting approximately 5-8% of the global population with limited treatment options. More than 80 autoimmune diseases have been identified and are considered a major global socioeconomic issue as they cause tremendous suffering to patients and lower their quality of life (1, 2). The higher proportion of publications retrieved from PubMed by using the keyword “autoimmune” compared to when using “immunology” indicates the increasing relative importance of autoimmune diseases between 2001 and 2019 (4). Although the pathogenesis of many of these diseases remains unclear, the identification of complex elements, including pathogenetic, genetic and environmental modulators, may lead to the development of new therapeutic strategies, earlier diagnostic capacity, and pinpoint autoimmune processes underlying such diseases (4, 5). However, the immunomodulatory drugs currently used to treat autoimmune diseases are characterized by broad-spectrum and non-specific properties, which may result in a range of side effects such as infections and malignant diseases (1). Therefore, there is an urgent need for precise early interventions. Recent data suggest that genomic information can provide a better understanding of the pathogenesis of autoimmune diseases and provide genetic susceptibility by identifying the genes and specific pathways that confer autoimmune disease. For example, the strongest and earliest genetic risk factors shared by autoimmune diseases identified to date are the HLA genes. These genes can predict the severity of the disease as well as suggest the responsiveness of the disease to biological therapies (6), thus providing refined management strategies. Based on this premise, Toll-like receptors (TLRs), a small family of proteins that are among the earliest determinants of immune activation, have become the focus of biomedical research. Studies have suggested that genetic variants of TLRs play a crucial role in different autoimmune diseases, such as type 1 diabetes mellitus (T1DM), Graves’ disease (GD), rheumatoid arthritis (RA), systemic lupus erythematosus (SLE), and multiple sclerosis (MS). This review provides a summary of the available data and draws appropriate conclusions.

## Gene Polymorphisms

The phenomenon that may result from point mutations is called gene polymorphisms, which is also known as single nucleotide polymorphism (SNP) and refers to single nucleotide differences in Some DNA sequences in the homologous interval (7). SNPs occur in > 1% of the general population, unlike other mutations that generally occur in ≤1% of individuals (8). In 2000, the first genetic variant of TLRs, a polymorphism of TLR4, was identified, in which two amino acid changes (D299G and T399I) have been reported to reduce the interaction of lipopolysaccharides with receptors (9), and patients are more susceptible to infection by gram-negative bacteria and sepsis (10).

Recent evidence identifies several SNPs in the genes encoding human TLRs (TLR1-10) and has shown that these SNPs can have functional effects on many human autoimmune diseases (11). In this review, we focus on SNPs of TLRs that have been previously studied and are involved in autoimmune diseases (12). Moreover, the impact of SNPs on disease pathogenesis and prognosis can be studied.

## Toll-Like Receptors

Regarding the detection of SNPs that are ubiquitous during the study of human systems, the research focus has shifted to the identification of SNPs at the signaling and receptor levels, particularly those associated with key receptors of innate immunity, i.e., TLRs. TLRs, named after Toll proteins in Drosophila melanogaster (13), are a family of inherent immune recognition receptors that detect microbial pathogens associated with molecular patterns to induce an immune response (14). Furthermore, TLRs is the first protein family that conforms to the characteristics of the pattern recognition receptor (PRRs) predicted by Janeway (15). As germline-encoded proteins, PRRs recognize conserved microbial products, also known as pathogen-associated molecular patterns (PAMPs), can induce host defense activities and stimulate immune responses (16). TLRs have been associated with a membrane protein involved in Drosophila embryogenesis and host defense (17). TLRs, in mammals, are synthesized in the endoplasmic reticulum (ER) after which they are transported to the plasma or endosomes (16).

Almost all organisms encode different numbers of TLRs, with humans and Drosophila encoding ten and nine TLRs, respectively (18, 19). The TLR in the plasma membrane in mammals is TLR4(primarily adapted to recognize LPSs and transduce LPS signals across the plasma membrane), which detects microbial cell surface components (16, 20); TLR5 recognize flagellin (21, 22), and TLRs 1, 2, and 6 detect bacterial lipoproteins (23–28); TLR3 specifically detects double-stranded RNA (dsRNA) (14), and TLR7 and 8 recognize single-stranded RNA (ssRNA) (29–31); TLR9 is a receptor for unmethylated CpG-containing ssDNA (32), while TLR13 recognizes bacterial ribosomal RNA (21). PAMP-PRR allows various members of the TLR family to recognize individual microbial cells through interactions (16).

**Figure 1:**
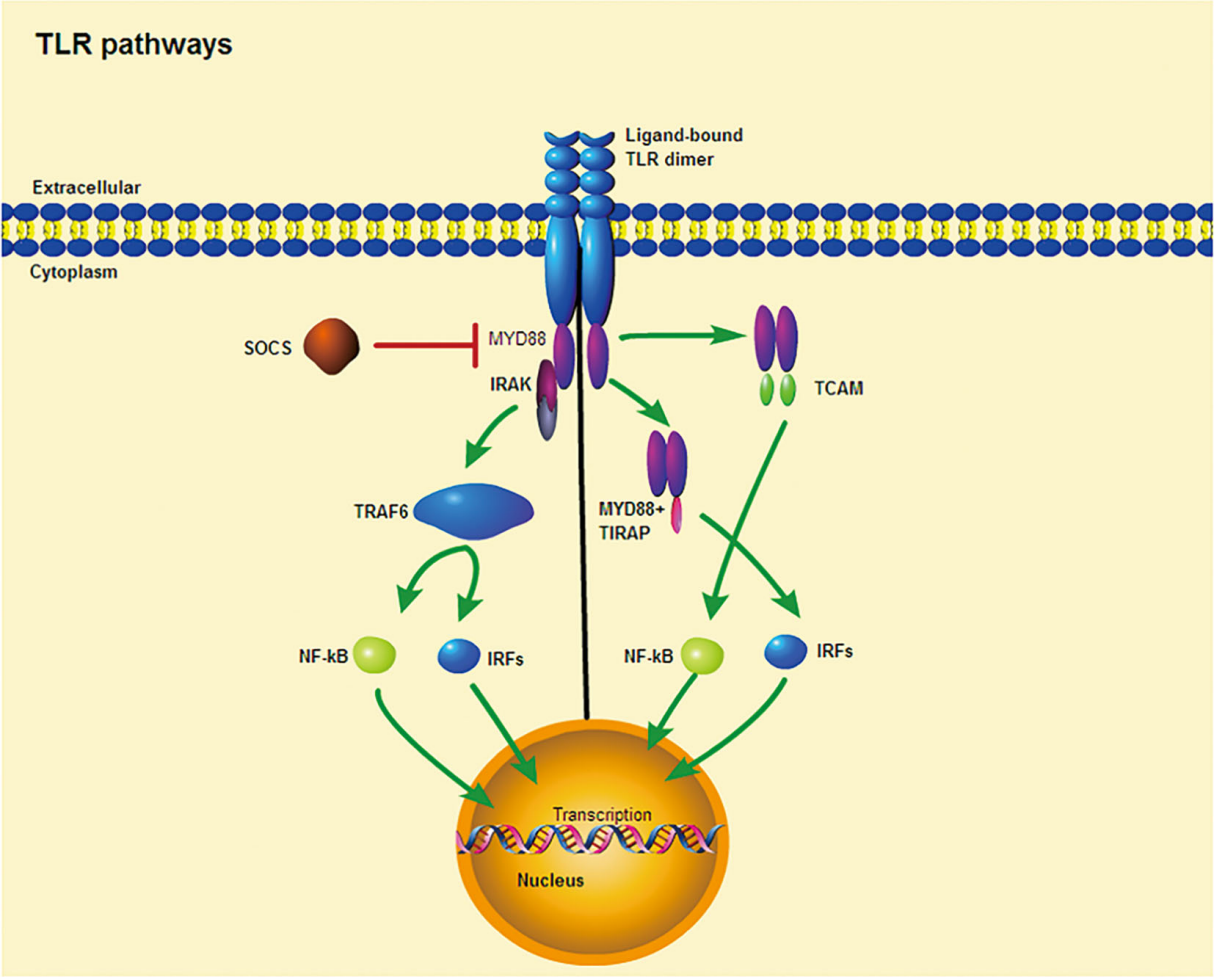
TLR signaling pathway. Ligand binding induces receptor dimerization and TLR interacts with TIR domain-containing adaptor proteins. (Left) TLR signaling occurs through a MYD88-dependent pathway. (Right) TLRs require other TIR domain-containing adaptors to induce cytokine expression, including TIRAP and TCAM. Purple ellipses denote conserved TIR domains. SOCS is a negative regulator of intracelluar cytokine signaling that can inhibit TLR responses.

All TLRs are composed of three primary regions: The extracellular domain is composed of tandem leucine-rich repeats (LRRs), the transmembrane helix, and an interleukin-1 receptor (TIR) domain/cytoplasmic Toll (33). The involvement of TLRs in the immune response is determined by the structure of the outer domain of the LRRs and their associated glycosylated superstructures (34). When bound to PAMPs, TLRs dimerize and transduce signals throughout the cytoplasm *via* the TIR domain (33). The receptor dimer is then recruited by different TLRs and activates different signaling pathways after interacting with the TIR domain-containing cytoplasm (35–37) (**Figure 1**). The TIR domains include myeloid differentiation factor 88 (MyD88), MyD88 aptamer-like protein (TIRAP or Mal), TIR domain-containing aptamer-inducible interferon-β (TRIF), and TRIF-related aptamer molecule (TRAM). MyD88, which is commonly used by all TLRs(except TLR3), possesses a death domain that recruits interleukin (IL)-1 receptor-related kinase (IRAK) family of serine-threonine kinases, which then initiate signaling pathways that ultimately activate transcription factors, most importantly nuclear factor kappa B (NF-κB) (35). Moreover, MyD88 is the first member of the TIR family to be identified and induces inflammatory cytokines by activating NF-κB and mitogen-activated protein kinases (MAPKs) (36). Similarly, IRAK4 is involved in the initiation of adaptive immunity through the activation of T cells by IL-1 (in T helper 17 [TH17] cells) and IL-18 (in TH1 cells) (38). In contrast, TRIF induces the production of type I interferons and inflammatory cytokines by recruiting TLR3 and TLR4 (37). TRAM and TIRAP act as binding adapters, recruiting TRIF to TLR4 and MyD88 to TLR2 and TLR4 (37). Thus, TLR4, the only receptor, activates both MyD88 and TRIF-dependent pathways. In summary, the TLR signaling pathway induces the production of inflammatory cytokines and type I interferons through two pathways, namely, MyD88-dependent and TRIF-dependent pathways (36). When inflammatory cytokine and type I interferon production are in balance, they play a key role in controlling tumor cell growth and avoiding autoimmune diseases (37), but when out of balance, they may influence the onset of autoimmune diseases.

In addition, the presence of TLRs gene polymorphism causes malfunction of certain crucial signaling pathways. TLRs play a crucial role in the production of autoreactive B and T cells, thereby increasing susceptibility to autoimmune diseases (39–43). In particular, there is evidence that TLRs play an important role in the pathogenesis of several autoimmune diseases, including T1DM, GD, RA, SLE, and MS (44–46). Thus, the study of TLRs gene polymorphism is important.

## Gene Polymorphisms and Toll-Like Receptors

The mechanisms by which TLR polymorphisms modify the functions of TLRs are not fully understood, and the latest studies suggest that SNPs affect receptor function through their effects on TLR expression, localization, trafficking and signaling. For example, I602S is a common TLR1 SNP that is associated with aberrant trafficking of the receptor to the cell surface (47). It has also been shown that the R753Q polymorphism alters the electrostatic potential of the TIR domain, thereby inhibiting TLR2-TLR6 dimerization, NF-kB activation and cytokine expression (48). The TLR4 D299G polymorphism inhibits the association between mutant receptors and adapters MyD88 and TRIF, suppressing activation of the transcription factor NF-kB (48, 49).

A comprehensive understanding of the molecular mechanisms by which various gene polymorphisms alter TLR function is expected to facilitate the development of more precise therapeutic strategies in autoimmune disease.

## Gene Polymorphisms and Autoimmune Diseases

### Type 1 Diabetes Mellitus

Type 1 diabetes, an autoimmune disease, is known as insulin-dependent diabetes which develops when pancreatic β-cells do not produce enough insulin and is accompanied by the destruction of these cells due to their own aggressive response (50). Type I diabetes (T1D) is an organ-specific autoimmune disease in which pancreatic beta cells are specifically destroyed by Th1 lymphocytes (51–53). Although the etiology of the disease is unknown, it is believed that a combination of environmental, genetic and stochastic factors contribute to the pathogenesis of T1D (51, 52). The prevalence of T1D varies widely across regions and ethnic groups. For example, its prevalence is 100-fold higher in some regions of Finland than in people living on the Korean Peninsula, which has stimulated interest in this area of genetic research (7).

One of the earliest studies focusing on the detection of SNPs associated with T1D in the TLR2 gene was conducted in 2004 on a Korean population (54). This study identified SNP rs3804100 (S450S, +1350). In addition, a similar study conducted in Norway obtained the same results. However, no such correlation was observed in the Spanish sample population (55).

Another study performed on T1D samples from the Basque Country identified different SNPs for TLR2 and TLR4 (56). However, in a Basque sample population, genetic association analysis suggested that there was no statistical association between TLR2 and TLR4 gene polymorphisms and T1DM (56). Other studies have shown that the above polymorphisms in TLR2 and TLR4 are not associated with T1D (57). There are several possible reasons for the observed discrepancy. First, the polymorphisms of TLR2 and TLR4, which have been studied, may not be etiologic variants. Additionally, it is possible that the low-passage polymorphisms in TLR2 and TLR4 are due to interactions of other genes or specific environmental features present in Basques. Furthermore, due to the similar distribution of alleles between the T1D group and affected family controls (AFBAC) in the Basque population, a larger sample size may be required to detect these associations.

Assmann et al. investigated whether the SNPs of TLR3 (rs11721827, rs13126816, rs5743313, rs7668666, and rs3775291) were associated with the development of T1DM (58). The authors demonstrated that TLR3 rs3775291 and rs13126816 polymorphisms were strongly associated with the risk of T1DM development (58). In the meantime, the rs5743313 and rs11721827 polymorphisms were associated with age at diagnosis of T1DM and poor glycemic control (58). In 2005, similar studies were conducted in South African T1DM patients to determine the association between this disease and possible polymorphisms of genes in TLR3. However, these studies, which recognized three SNPs(rs5743313 [2593 C/T], 2590 G/C, and rs5743315 [2642 C/A]) presented ambiguous results (7, 59). The study also reports that the latest discovered genetic polymorphisms were not detected in black South Africans (59). This may indicate that South Africans have race-specific genes, implying that these three polymorphisms may still be associated with T1DM.

In 2015, a total of 28 SNPs associated with TLR1, TLR2, TLR3, TLR4, TLR5, TLR6, TLR8, and TLR9 were genotyped in China, many of which were not studied in previous genome-wide association studies (GWAS), and showed for the first time that SNPs of TLR1 (rs5743612, -626; rs4833095, -1017) may play a crucial role in the etiology of T1DM (55). Overall, the data obtained from previous studies do not conclusively confirm a correlation between polymorphisms of TLRs and T1DM.

Therefore, future studies must expand the sample size of patients and controls. Due to complex genetic factors and several external factors, T1DM remains “a geneticist’s nightmare”, as noted by James Neel (60, 61). Nevertheless, although inconclusive, TLR genes remain possible candidates for T1DM.

### Graves’ Disease

Graves’ disease (GD) is a hyperthyroidism associated with organ-specific autoimmune inflammation, which is characterized by an overactive and enlarged thyroid gland (Graves’ hyperthyroidism), eye abnormalities (Graves’ orbitopathy; GO), and localized dermatosis (anterior tibial myxedema; PTM) (62). The disease can occur at any age, but it is most common in women of childbearing age and is the most common cause of hyperthyroidism in the world (63, 64). Graves’ hyperthyroidism was initially thought to be caused by excessive secretion of thyroid-stimulating hormone (TSH) by the pituitary gland, but the discovery of autoantibodies against TSH receptor (TSHR) in 1956 has established GD as an autoimmune disease (62, 65).

Currently, GD is considered to be highly heritable and polygenic in nature, and is most likely caused by a combination of susceptibility genes and environmental triggers, including Yersinia enterocolitica (65). A study in 2013 provided a hypothesis for molecular mimicry in GD, where early B-cell precursors are amplified by Y. enterica porins and achieve somatic hypermutation to induce cross-immune responses to TSHR (65). The etiology of GD remains unclear; however, GD appears to be multipathogenic in nature, hence, attempts have been made to identify the genetic factors that influence its etiology, particularly the discovery of relevant polymorphisms.

The association of polymorphisms in the TLR gene with GD was studied in a Cantonese sample from China (66). Three SNPs, rs5744174 for TLR5, rs5743565 for TLR1, and rs179010 for TLR7, were conducted in 332 patients with GD and 351 unrelated controls (7, 66). According to the dominant model, the coding region of these three SNPs were protective in females (66). Several theories have been proposed as to why these polymorphisms play a protective role. First, TIR domain-containing adapter proteins initiate adaptive immune responses by releasing a cascade of signals and influencing TLR signaling (7). Second, SNP rs5744174 attenuates the flagellin response to cytokines, thereby reducing susceptibility to bacterial infection (7). Third, these three SNPs can affect translation, reduce TLR protein expression, decrease autoantibody or cytokine production, and regulate the immune response to ligands, which may explain why GD risk can be reduced in the presence of these specific gene polymorphisms (7).

In 2017, a study investigated on the association of polymorphisms of TLR4 and TLR9 with autoimmune thyroid disease in Korean pediatric patients found that women with GD exhibited higher frequencies of the TLR4 rs10759932 T allele and rs1927911 CC genotype compared to controls (67). These results suggest that polymorphisms in TLR4 is likely to affect negatively in women with GD.

Tomer et al. found that the SNP rs2284720 (within intron 1) in the TSHR gene was strongly associated with GD (68). In addition, genetic variation in the extracellular A subunit of TSHR (capable of shedding from the cell surface) may alter its expression and/or shedding capacity, thus affecting the circulating level of this autoantigen and the strength of the autoimmune response (69). However, the exact mechanism of this process remains unknown.

Currently, although the data on GD suggest that patients with identified TLR gene polymorphisms have disease susceptibility, they are not sufficient to thoroughly assess the molecular mechanisms underlying the aforementioned susceptibility. Moreover, most of these studies were conducted using small sample sizes involving individuals from a single ethnic group, which significantly reduces the reliability of the results obtained regarding the risk of disease development.

### Systemic Lupus Erythematosus

Systemic lupus erythematosus, an autoimmune disease, involves multiple systems which characterized by loss of self-tolerance, formation of nuclear autoantigens and production of immune complexes (70). SLE has various clinical symptoms that can involve one or more organs such as the skin, kidneys, joints and nervous system, and is characterized by a chronic or relapsing-remitting course (70). It took nearly a century to recognize that lupus erythematosus, initially considered as a skin disease, is a systemic disease whose pathogenesis involves an abnormal autoimmune response (71). Vital organs and tissues, such as the brain, blood, and kidneys are affected in many patients, most of them in women of reproductive age (71). The incidence of SLE ranges from 20 to 150 cases per 100,000 individuals (71), and the 10-year survival rate has significantly increased by more than 70% over the past 50 years, largely due to increased awareness of the disease, widespread and informed use of immunosuppressive agents, and more effective treatment of infections, which are the leading cause of death in patients (72). The diversity of SLE clinical features matches the complexity of its pathogenesis, which involve genetic, hormonal and environmental factors (73).

Human plasmacytoid dendritic cells (pDCs) are a key factor in the pathogenesis of autoimmune diseases (74). In SLE, pDCs can induce cytokines and chemokines production through the combination between TLRs and Fc-γreceptors (Fc-γ-Rs) (75, 76). Several studies have suggested that TLRs are involved in the complex pathogenesis of SLE. For example, one study found that increased expression of TLR9 mRNA at the onset of SLE was associated with a poorer prognosis during 2-year follow-up.

Other studies have also shown that SNPs of TLRs are associated with SLE. SNPs of TLR7 (rs3853839), TLR8 (rs3764880) and TLR9 (rs351240) increase the risk of SLE in Asian populations (77, 78). In Danish samples, SNPs for TLR3 (rs3775291), TLR8 (rs3764879) and TLR9 (rs352143) were associated with SLE (79). These results suggest that genetic variants in certain TLRs are associated with the pathogenesis of SLE and are related to the susceptibility and clinical phenotype of SLE.

One study investigated SNPs for TLR pathway-associated proteins, including IRAK-M (interleukin receptor-associated kinase-M) (rs11465955, rs1624395, rs1152888, and rs1370128) and SIGIRR (single immunoglobulin IL1-1R-related molecules) (rs3210908). IRAK-M and SIGIRR have a negative regulation of TLR signaling (80, 81). SIGIRR is a member of the IL-1R family which inhibits MyD88-dependent TLR and IL-1R signaling (82, 83). Studies in SIGIRR-deficient mice have shown the importance of SIGIRR in enhancing B-cell activation and proliferation, including autoantibody production against multinucleated lupus autoantigens (84, 85). Furthermore, IRAK-M is located on chromosome 12q14, which was identified as an SLE susceptibility locus in a GWAS (85). However, the results of this study suggest that the studied variants in the IRAK-M and SIGIRR genes do not influence SLE susceptibility in populations of European descent (86). Although it remains unclear whether IRAK-M and SIGIRR genes are functionally relevant to the pathogenesis of SLE, animal models have yielded promising results. Therefore, further genetic studies in populations of different ethnic backgrounds are warranted.

### Rheumatoid Arthritis

Rheumatoid arthritis is a common inflammatory systemic autoimmune disease with a global prevalence of approximately 1% in the adult population and is characterized by painful and swollen joints that severely affect motor function, as well as quality of life (87). The expression levels of various TLR isoforms were higher in the synovial tissue of RA patients compared to healthy controls (88–90). Ligands for TLR3 (host-derived RNA) and TLR4 (HSPB8) are also abundant in the blood and synovial joints of RA patients (88, 89, 91). Furthermore, TLR4-mediated dendritic cells stimulation resulted in significantly higher cytokines concentrations in RA patients than in controls, which further supports a deranged TLR response in RA (92).

In the Turkish population, the TLR9 rs187084 allele variant TT increases susceptibility to RA, although no such association was found in the Dutch, Swedish, or British cohorts (92–94). Furthermore, in a cohort study of 319 Europeans, the SNP rs5741883 of TLR8 was associated with RA-associated autoantibody positivity (92). In a study of a British Caucasian sample, SNP rs7514863 for the TRAF5 gene was observed in a whole cohort of 1273 RA patients compared to 2463 healthy controls (95). In a large group of patients and controls, these findings provide a larger platform for strong candidate RA susceptibility genes. However, further studies are warranted to demonstrate the role of this variant or variants in the development of RA or in more autoimmune diseases.

### Multiple Sclerosis

Multiple sclerosis is a chronic inflammatory demyelinating disease of the central nervous system, and immune responses are involved in its pathogenesis, resulting in slowing or stopping of nerve impulses due to damage or complete absence of the protective layer or myelin sheath around nerve cells. The most frequent cumulative sites of the disease are the periventricular region, proximal cortex, optic nerve, spinal cord, brainstem and cerebellum (96). This leads to a variety of clinical symptoms, including limb weakness, sensory abnormalities, ocular symptoms, ataxia, psychiatric symptoms as well as other neuronal complications (97). In several countries, MS is a major cause of disability in young individuals. Approximately 2.3 million individuals suffer from MS worldwide (98). MS is associated with high social and economic burden and increases over time. The economic burden associated with MS in Europe was estimated to be approximately 14.6 billion euros in 2010, and the United States bore the associated costs of approximately $4.3 billion in 2013 (99). Although the etiology of MS is currently unknown, many disease-related risk factors have been identified. Among them, the most identified risk factors include low vitamin D levels, smoking and infection with Epstein-Barr virus (100, 101). The genetic risk factors for MS include genes associated with the immune system, such as the HLA gene, IL2 and IL7R (100).

In addition, each TLR responds specifically to the molecular pattern associated with the pathogen (102). However, TLR signaling must be regulated in some way to prevent unnecessary damage to the host (102). Suppressor of cytokine signaling (SOCS)-1, negative regulator, inhibits TLR responses. SOCS proteins, particularly SOCS-1 and SOCS-3, are expressed in immune cells and cells of the central nervous system (103). In addition to its immunomodulatory function in the immune system, the SOCS family can activate immune cells such as microglia and macrophages. A moderate negative correlation has been reported between SOCS-1 and SOCS-3 expression. Compared to healthy controls, MS patients had lower levels of SOCS-1 transcripts and higher levels of SOCS-3 transcripts in peripheral blood leukocytes (104). SOCS-1 and SOCS-3 can regulate Th17 cell differentiation by acting on cells of the innate and adaptive immune system (105, 106). Th17, notably, has an important role in the pathogenesis of MS. Furthermore, statins affect the expression of SOCS-3, which is essential for the inhibition of Th17 cell differentiation, and have a therapeutic effect in relapsing-remitting MS (106).

The SOCS-1 variant of the SNP rs243324 is considered a risk factor for MS in the Spanish population (5). In addition, its T allele increases the risk of RR MS (107). Another study proposed two possible genetic markers, rs352162 and rs187084, that could be associated with gender differences observed in multiple sclerosis (108).  However, in 2008, Carvalho et al. Has observed no significant association between MS and disease severity, time of onset, or disease subtype in the Portuguese population (109). Therefore, a larger cohort study is warranted to confirm these results.

NF-κB is a key downstream component of the TLR signaling pathway. Furthermore, NF-κB-mediated upregulation of adhesion molecules and cytokines leads to an enhanced inflammatory response (110). The DNA-binding activity of NF-κB is increased in macrophages of MS patients after stimulation by relevant TLR ligands and cytokines (111). NF-κB activation may indicate myelin damage in MS lesions through an inflammatory response (5). TLR ligands are already identified as T cell-specific promoters in MS (5). In addition, TLRs play different roles in axonal pathway formation, dorsoventral patterning, and cell fate determination. However, there are no studies on the development of SNPs and MS for the NF-κB gene; hence, further investigations are warranted.

## Discussion

SNPs of TLRs are involved in various autoimmune diseases (**Table 1**). As summarized in the **Table 1** and from an epidemiological analysis, there is no doubt that gene polymorphisms affect the development of autoimmune diseases. But how these polymorphisms contribute to the development of the autoimmune disease is not fully understood. Importantly, genetic polymorphisms are observed in multiple TLRs which greatly increases the size and complexity of the study. In turn, this is probably the essential reason for the lack of sufficient confirmed data to develop a comprehensive picture of the real associations between polymorphisms and autoimmune diseases.

**Table 1:** Association between TLRs SNPs and autoimmune diseases.

Disease	Gene	Rs#	Sample population	Number of patients	Number of controls	OR	p valune	Ref
Type 1 Diabetes Mellitus	TLR1	rs4833095	Chinese Han population	429	300	2.22	0.03	(53)
		rs5743612	Chinese Han population	429	300	1.98	0.01	(53)
	TLR2	rs3804100	Korean population	407	1142	1.7	≤0.05	(52)
	TLR3	rs5743313	South African Blacks	79	74	1.1	0.042	(57)
		rs5743315	South African Blacks	79	74	0.68	0.03	(57)
		rs3775291	Brazil	449	507	2.3	0.004	(56)
		rs13126816	Brazil	449	507	2.1	10^-4	(56)
		rs5743313	Brazil	449	507	NO	0.561	(56)
		rs11721827	Brazil	449	507	NO	0.053	(56)
Graves' Disease	TLR5	rs5744174	Chinese Cantonese population	332	351	0.63	0.003	(64)
	TLR1	rs5743565	Chinese Cantonese population	332	351	0.72	0.005	(64)
	TLR7	rs179010	Chinese Cantonese population	332	351	0.64	0.004	(64)
	TLR4	rs10759932	Korean	60	183	2.06	0.015	(65)
		rs1927911	Korean	60	183	1.96	0.009	(65)
	TSHR	rs2284720	North American Caucasian	225	183	1.6	0.03	(66)
Systemic Lupus Erythematosus	TLR9	rs351240	Chinese Han population	430	424	NO	≤0.05	(78)
	TLR7	rs3853839	Taiwanese population	795	1162	2.82	0.01	(79)
	TLR8	rs3764880	Taiwanese population	795	1162	2.91	0.0173	(79)
	TLR3	rs3775291	Danish	143	432	1.53	0.006	(80)
	TLR8	rs3764879	Danish	143	432	1.47	0.013	(80)
	TLR9	rs352143	Danish	143	432	1.46	< 0.02	(80)
Rheumatoid Arthritis	TLR8	rs5741883	European population	319	319	0.88	0.02	(93)
	TLR9	rs187084	Swedish patients	2158	1068	NO	NS	(94)
	TLR9	rs187084	Dutch	378	294	0.92	0.48	(94)
	TLR9	rs187084	UK	406	430	NO	NS	(94)
	TLR9	rs187084	Turkish population	100	100	1.85	0.01	(95)
	TRAF5	rs7514863	British	1273	2463	1.2	0.005	(96)
Multiple Sclerosis	SOCS	rs243324	Spain population	3919	4003	1.13	0.00006	(108)
	TLR9	rs5743836	Portuguese population	165	165	NO	0.274	(110)
	TLR9	rs352162	white Spanish individuals	574	807	0.75	0.024	(109)
	TLR9	rs187084	white Spanish individuals	574	807	1.66	0.028	(109)

The following are possible mechanisms for the interaction of TLRs, SNPs and autoimmune diseases. Since human evolution has developed, the positive selection pressure may provide a plausible explanation for the formation of TLR polymorphisms and their involvement in the pathogenesis of autoimmune diseases. Some TLR gene polymorphisms may be the result of positive selection, and such polymorphisms may have protective or deleterious effects against infections caused by certain pathogens, but also affect the development of autoimmune diseases. For example, TLR polymorphisms can render TLR5 functionally deficient. TLR5 recognizes flagellin, an important PAMP (flagellin bacterium) (21, 22). Mutations in TLR5 (Arg392 replaced by a stop codon) result in TLR failure to recognize flagellin (112). Interestingly, this polymorphism is present in about 10% of the European population and this genetic polymorphism can increase susceptibility to Legionella pneumophila infection, but the mutation has a protective effect against SLE (113). Genetic variation, a driver of human evolution and natural selection, has enabled modern humans to respond to a wide range of infectious agents. However, it may also predispose humans to immune response imbalances that influence susceptibility to autoimmune diseases.

Furthermore, immunohistochemical studies have shown that TLR3 protein is overexpressed in thyroid cells in patients with Hashimoto’s thyroiditis, but not in thyroid cells in the normal population (114). Future research could be directed to investigate whether TLR gene polymorphisms could reduce TLR3 expression levels or alter the functions of TLRs, thus bringing new targeted therapies for patients with Hashimoto’s thyroiditis.

Research related to genetics and immunity is an emerging field, and in-depth study of Toll-like receptor gene polymorphisms in autoimmune diseases will better address clinical issues. For example, SNPs are important for genome-wide association studies (GWAS) (7), the main purpose of which is to identify genetic risk factors, and will help to reasonably predict susceptibility to autoimmune diseases. Further research in this area can provide more information about the effects of SNPs on genes, genomic risk markers used to predict and prevent disease, and will also help to identify new approaches to diagnose, treat and develop prevention of autoimmune diseases using gene therapy.

Moreover, the prevalence of co-existing autoimmune diseases, such as that of autoimmune encephalitis and myelin oligodendroglial glycoprotein antibody disease, has increased in recent years, as indicated by a recent study (115). In addition, another study has demonstrated that the HLA class II allele DRB1*16:02 is involved in the pathogenesis of autoimmune encephalitis and reported an association between specific HLA class II alleles and autoimmune encephalitis for the first time, thus providing new insights into the pathogenesis of this disease (116). Since a clear relationship has been established, future studies should be aimed at determine whether the HLA class II allele DRB1*16:02 is involved in the pathogenesis of both autoimmune encephalitis and myelin oligodendroglial glycoprotein antibody disease.

In 2015, a study conducted in China reviewed the clinical data of 616 Chinese patients admitted to Peking Union Medical College Hospital (PUMCH) with a diagnosis of portosystemic shunt (PSS) between January 1985 and December 2013. Of the 616 patients, 43 developed neuromyelitis optica spectrum disorder (NMOSD) (117). Additionally, PSS and NMOSD have been found to commonly coexist in the Asian population. Our team will also continue to investigate in depth whether there is a particular immune-related gene polymorphism that affects the susceptibility to PSS and NMOSD.

Although current genetic studies of immune-related diseases are emerging, several other related genes and pathogenic variants remain to be investigated. However, the concept of common genetic pathways offers a novel and effective approach to enhance the discovery of the full range of susceptibility genes, as genetic resources can be shared, rather than targeting only a single disease (118). This would thus provide new strategies for the treatment of autoimmune diseases.

It is important to consider how future studies of this nature might be conducted. Epidemiological studies using large population biobank enable us to track individuals with certain genetic risks over a long time and to monitor their environmental exposure and disease outcomes (118). In addition, such studies can improve our understanding of the sequence and timing of multiple immune disease onsets in the same individual. We believe that knowledge regarding common genetic pathways can be used to make predictions about disease progression, which can be useful in clinical practice.

## Conclusions

Numerous studies have highlighted the important role of TLRs in autoimmune diseases. Moreover, SOCS and TRAF proteins are considered to be major regulators of the TLR pathway. The expression of these proteins as well as the elucidation of SNPs may explain the pathological mechanisms underlying autoimmune diseases. However, these findings should be interpreted with caution, since several other factors may contribute to the observed variability and discrepancies. Studies remain limited to relatively small sample sizes and population stratification (especially in mixed populations). Moreover, the impact of geographical and environmental factors on the disease should be considered. Furthermore, deeper knowledge of TLR families could help the development of more targeted therapies for autoimmune diseases, such as the use of statins for the treatment of MS. In addition, genetic profiling can help to better identify individuals at risk for autoimmune disease, which will facilitate timely diagnosis and treatment. However, some of the same SNPs of TLRs can exist for two or more autoimmune diseases, suggesting that more in-depth studies are warranted in this area.

## Author Contributions

WL designed the review. JiL, CW, and JuL collected materials. YZ drafted the manuscript. WL takes responsibility for the integrity of the work from its inception to publishing. All authors contributed to the article and approved the submitted version.

## Conflict of Interest

The authors declare that the research was conducted in the absence of any commercial or financial relationships that could be construed as a potential conflict of interest.
